# Putting the cart before the horse: mixed-methods participatory investigation of working equid harnessing practices in three selected towns of the Oromia national regional state in Ethiopia

**DOI:** 10.1186/s12917-024-03967-3

**Published:** 2024-03-22

**Authors:** Mathilde S. Merridale-Punter, Belay Elias, Abel L. Wodajo, Charles M. El-Hage, Hanna Zewdu, Reta Tesfaye, Gizachew Hailegebreal, Teshale Sori, Anke K. Wiethoelter, Peta L. Hitchens

**Affiliations:** 1 Melbourne Veterinary School, Faculty of Science, University of Melbourne, Parkville, VIC 3010 Australia; 2 College of Veterinary Medicine and Agriculture, Addis Ababa University, P.O. Box 34, Bishoftu, Ethiopia; 3 Faculty of Veterinary Medicine, Hawassa University, P.O. Box 05, Hawassa, Ethiopia; 4 Equine Centre, Melbourne Veterinary School, University of Melbourne, 250 Princes Hwy, Werribee, VIC 3030 Australia

**Keywords:** Harness, Cart, Horse, Donkey, Mixed-methods

## Abstract

**Background:**

Millions of working equids provide socio-economic support for many low-income communities worldwide. With the prevalence of harness-related wounds reported as higher than 60%, this study aims to describe the equipment used by working equids in three locations of the Ethiopian national regional state of Oromia (Fiche, Bishoftu and Shashamene), and the attitudes and practices of equid owners, users and harness makers regarding work equipment. This mixed-methods study consists of cross-sectional surveying of working equids used for taxi or transport of goods or water, as well as cart-driver questionnaires and focus groups (FG) with working equid stakeholders. Activities conducted with FG included participatory ranking of equipment attributes and equipment drawing exercises. Indicators of equipment design and assembly, as well as cart-driver attitudes and practices were described quantitatively. Associations between equipment characteristics and species, work-type and cart-driver indicators were investigated through univariable logistic regression models, whereas focus group discussions were transcribed and analysed thematically.

**Results:**

In total, 368 working equid surveys and cart-driver questionnaires were completed and 87 participants took part in nine FG. Equipment composition and characteristics differed considerably from ideal animal draught and harnessing principles described in the literature, with none of the observed harnesses adhering to all principles and thus not considered fully adequate. Various harness compositions were used, with only saddles and breast collars present in all. Donkey equipment had fewer components than that of horses, such as swingle trees (OR 0.02; 95% CI 0.01–0.06; *p* < 0.001) or girths (OR 0.09; 95%CI 0.02–0.4; *p* = 0.002). Horse equipment was more likely to have functional elements such as breeching (OR 7.8; 95% CI 2.9–20.9; *p* < 0.001). Of all equipment attributes, FG participants ranked cost, design and impact on animal wellbeing as having the highest importance. Thematic analysis identified motivations and priorities regarding equipment; awareness and knowledge of design and function; barriers to using ideal equipment; and consequences of equipment design and practices as key themes.

**Conclusions:**

Various weaknesses of design, assembly and use of equid work equipment were identified. Promoting behavioural change through awareness and training could lead to a sustainable improvement of work equipment quality, access, and prevention of equipment-related problems.

**Supplementary Information:**

The online version contains supplementary material available at 10.1186/s12917-024-03967-3.

## Introduction

An estimated 36 million equids support the 38 lowest-income countries worldwide [1]. Ethiopia holds the largest equid population in Africa, where 11 to 12 million equids [1, 2] are used mainly for transport, draught and pack work [2–4]. These working equids are often the primary source of household income and play an essential role in supporting the wider community through their work in agriculture, supply chains, and transport of people, goods and water [3, 5].

The labour working equids are required to do typically involves the use of work equipment, and as these animals frequently work for several hours a day, often seven days per week [6, 7], equipment becomes an important part of their lives. In the case of riding- or pack-saddles, the equipment allows for the loading of people or cargo directly onto the animal, whereas in the case of animals used for cart-pulling or agricultural work, equipment requires a harness which is then connected to a cart or agricultural implements respectively. Although there are various harness types and designs [8–12], basic animal draught and harnessing principles should be respected where working equids are used for cart work, to efficiently capture animal draught power while ensuring the animal is able to work comfortably and safely [8, 13–16]. These include the principles of steering (providing direction, usually achieved through a bridle or a bit and reins), transmission (where animal power is transmitted to the cart through a collar attached to traces in order to move it), weight distribution (where the weight of the cart is distributed across the animal’s back through a padded cart-saddle), and braking (where a breeching system is used around or behind the hindquarters, attaching to the shafts of the cart taking its weight when reversing or going downhill) [8, 9, 13, 15]. To enable these animal draught principles, specific components should be present in a harness, and the way in which they are assembled together and fitted to the animal are also of critical importance to achieve their intended function [8, 9, 13–18].

Equipment design influences animal comfort and efficiency of work [12], and should be suitable for both the animal, the user and the specific type of work intended [8, 14, 16, 18]. However, equipment-related wounds are common in working equids worldwide [19–24] and reported in over 60% of animals in some parts of Ethiopia [25, 26], raising concerns about the adequacy of the work equipment and the work practices used [6, 19, 27]. Inadequate work equipment has also been associated with lameness [6] and other indicators of poor welfare [19, 24]. However, despite recognition that the equipment is often not fit for purpose [6, 19], the extent and degree of these inadequacies is not well reported. Critically, the drivers and barriers for access and usage of work equipment that is fit for purpose are not well understood.

To this end, the aims of this study were to (1) describe the work equipment used by working equids in three Ethiopian locations; (2) understand the implications of current harnessing practices to both animals and the community; and (3) describe the knowledge, attitudes and practices (KAP) of working equid users in regard to work equipment. A greater understanding of cart and harness design characteristics as well as motivating influences around work equipment choices can be applied towards devising evidence-based and problem-specific recommendations to owners and cart and harness manufacturers. It is anticipated that this may facilitate the development of more sustainable strategies for the prevention of equipment-related wounds and enhance the overall welfare of these equids.

## Methods

### Study design

**Fig. 1:**
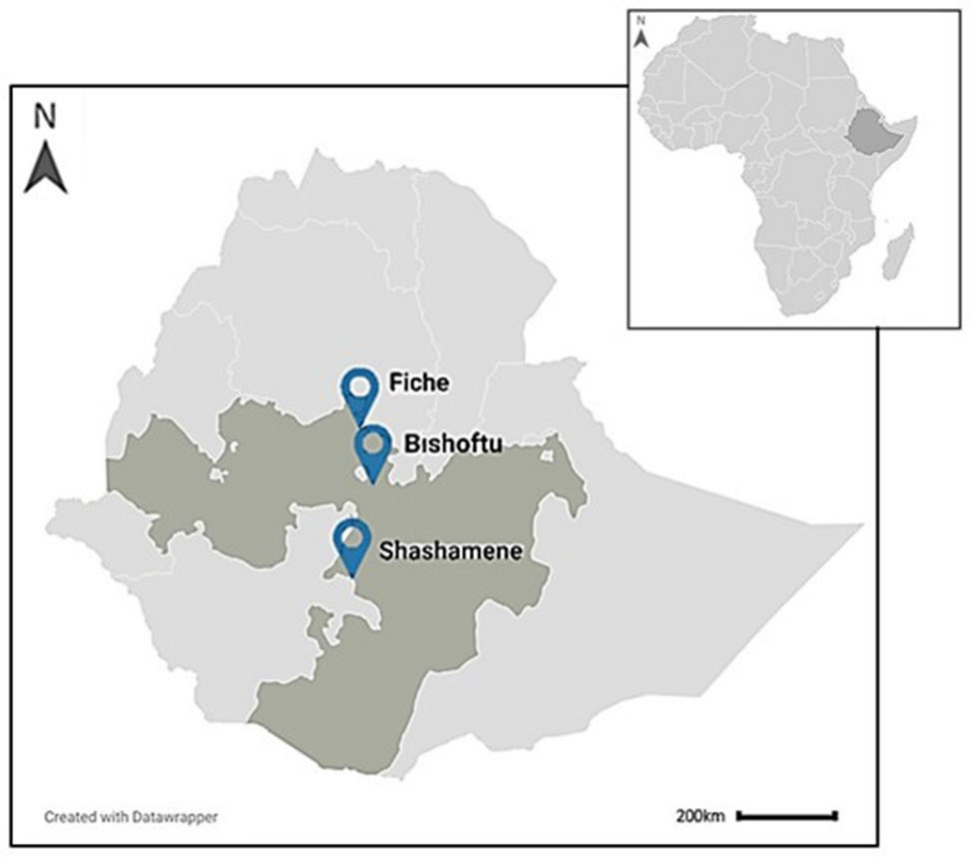
Map of study locations to investigate harnessing equipment and practices in Ethiopian working equids. The close-up map of Ethiopia indicates the Oromia national regional state in dark grey and the three study locations: Fiche, Bishoftu and Shashamene. The map insert (top-right) shows the African continent with the country of Ethiopia highlighted in dark grey. Figure created in Datawrapper [28]

Data collection for this study took place in Ethiopia between February and April 2022, in three towns of different administrative zones of the Oromia national regional state: Bishoftu (East Shewa Zone), Fiche (North Shewa Zone) and Shashamene (West Arsi Zone) (Fig. 1).

Bishoftu and Fiche were selected based on the extensive use of cart-horses in these towns, while Shashamene was selected due to its predominance of cart-donkeys (local knowledge provided by HZ and RT). A working equid was considered as a horse, donkey or mule used for cart-work, transporting people, goods, water or other items through a cart and harness hitching system.

Data was obtained through a mixed-methods approach: (1) quantitative data was collected through a cross-sectional survey of equids used for cart work and questionnaire to their respective cart-drivers; and (2) qualitative data was obtained through a series of focus groups (FG) with working equid stakeholders.

Time-loss compensation was provided in the form of a pre-paid equid feed voucher, or equivalent cash compensation for participants who did not own an equid (such as harness-makers). All methods were carried out in accordance with relevant guidelines and regulations for conducting research in Australia and Ethiopia.

### Quantitative data collection and analysis

**Fig. 2:**
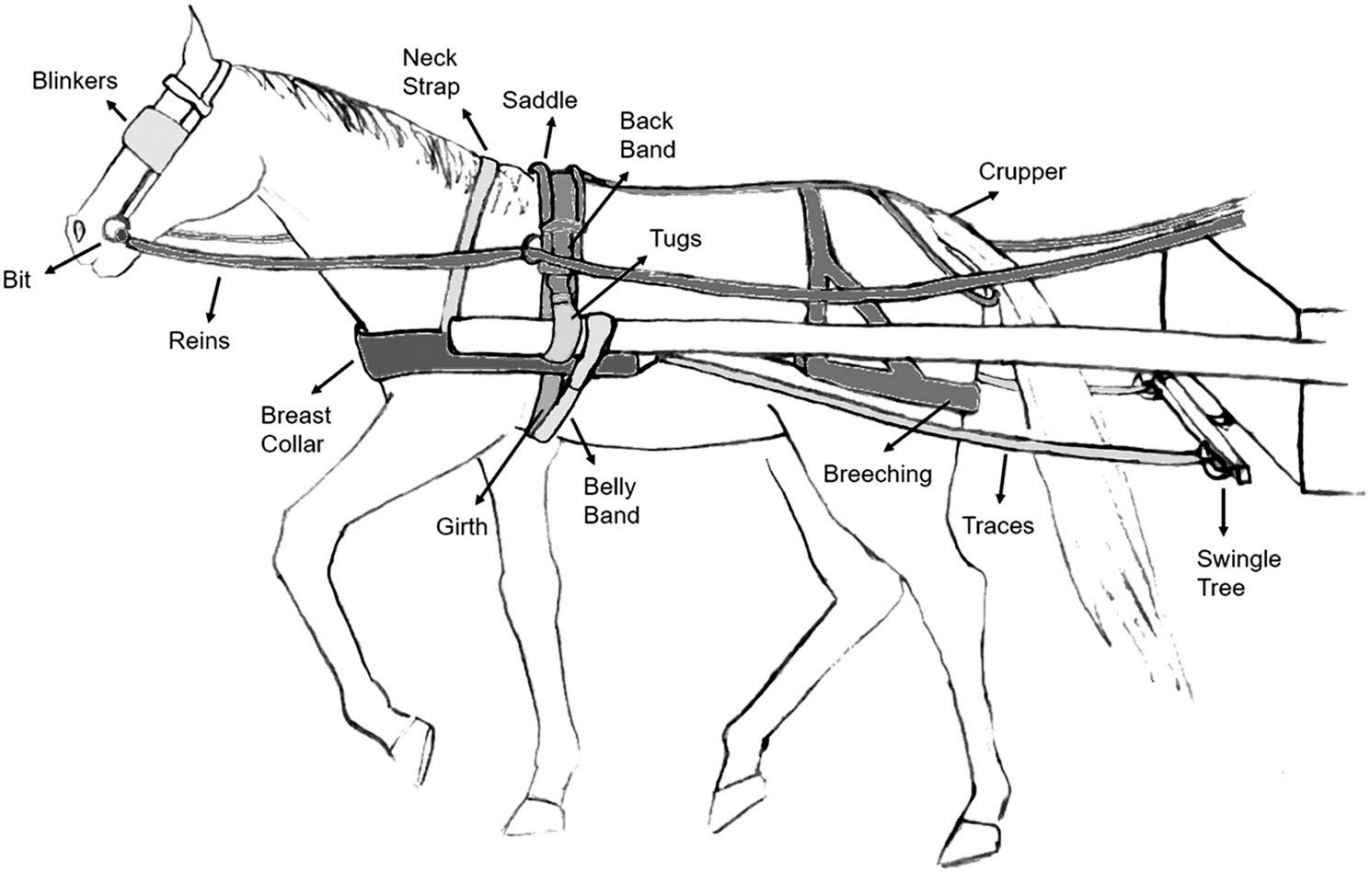
Illustration of basic components of a typical breast collar harness, in a study investigating harnessing equipment and practices in Ethiopian working equids. Components illustrated include a bit, blinkers, reins, collar, neck-strap, traces, saddle, backband, tugs, girth, belly band, breeching, crupper and swingle tree [11, 13, 18]. A general illustration of typical harness design is represented, without dismissing other appropriate harness designs, additional components or variations according to species, animal, vehicle or work characteristics. Illustration created by N. Galinelli and M. Merridale-Punter

As no data on the prevalence of adequate equipment in the study locations has been published to date, sample size calculation was based on the expected prevalence of harness-related wounds as an indicator of equipment quality. The prevalence of harness-related wounds in Shashamene and Bishoftu has been reported at 17% and 18% respectively [22, 29], and thus a required sample size of 369 equids and drivers was estimated, based on an expected prevalence of harness-related wounds of 20% [30], a desired confidence of 95%, and an absolute precision of 5% and inflating by a design effect of 1.5 to account for clustering [31]. Two data collection sheets were created: a survey of animal welfare indicators [32] and work equipment indicators for each equid, and a questionnaire for the corresponding cart-driver (Supplement 1). Work equipment characteristics surveyed focused on the presence or absence of equipment components [8, 11, 13, 15, 17] such as bit, blinkers, reins, collar, neck-strap, traces, swingle tree, saddle, saddle padding, backband, tugs, girth, belly band, breeching and crupper (Fig. 2); cart characteristics such as number of wheels, tyre inflation, axel balance as well as indicators of equipment quality like fit and hitching. Cart-driver questionnaires collected data on demographics, professional training, choice of equipment, equipment maintenance, harness assembly and hitching practices, and attitudes regarding the interaction between the animal and its equipment (Supplement 1). Animals and their cart-drivers were recruited at different cart stations (holding areas where cart-drivers collect goods or taxi customers) selected by convenience within each study region. A random systematic sampling frame was applied within each station to reduce selection bias, where cart-drivers were assigned a number and every-other number was selected to participate in the study. All data was collected in local language and translated to English by the same two trained assessors (AL and BE).

Survey and questionnaire data were entered into the secure, web-based electronic data capture tool REDCap [33, 34], hosted at Melbourne Veterinary School. Descriptive and inferential statistics were performed using R software (2021 R Core Team [35]). Where the functionality of a given harness component was dependent on the characteristics of multiple interacting elements recorded individually, additional variables were generated to represent their functionality as a unit: functional swingle tree (where a swingle tree is present, moves freely and traces attach to the swingle tree); functional saddle (a saddle is present, is adequately positioned, has wide pressure points, has gullet, has padding and is secured tightly to the animal); functional traction system (a collar is present, is adequately fitted over the point of draught and connects to freely moving traces that attach to a functional swingle tree); functional breeching (breeching is present and is assembled correctly as either true or false breeching); functional weight distribution (functional saddle as well as presence of a belly band, back band, tugs, and shafts passing through the centre of gravity); and functional steering (presence of bit with adequate size and condition, connected to freely moving reins) [14, 15, 18]. Harness contact surface materials were recorded individually for each harness component and subsequently classified as either breathable (cloth, canvas, leather) or non-breathable (plastics, nylon, rubber, rope) materials. A fully adequate harness was considered as a harness that simultaneously included functional traction, functional weight distribution, functional breeching and functional steering [15], and that was made from atraumatic, breathable materials [11, 13, 18, 36]. The user’s estimation of the proportion of different work types performed by the animal was recorded - taxi, transport of goods, transport of water or other. Work types were then categorised according to the work performed in highest proportion, and where ‘mixed’ was denoted if the animal performed equal proportions of different work types. When asked where they have learnt about work equipment, participants were allowed to select multiple sources, however, for the purposes of analysis these were categorised into three training sources: ‘Intuition and Observation’ if learning was entirely independent, ‘Other Drivers’ for respondents also learning from peers; and ‘Harness Maker’ for any combination of sources that included a harness maker.

Univariable logistic regression models were generated to test for associations between the presence of individual equipment components or characteristics (outcome), and species, work type and questionnaire variables relating to equipment and training source, income comfort level, driver experience and views on equipment efficiency (predictors). To reduce the risk of type I errors, a false discovery rate (FDR) correction for multiple comparisons was applied to all regression models using the Benjamini-Hochberg adjustment method [37]. Odds ratios (OR) and 95% confidence intervals (95% CI) are presented, as well as unadjusted and adjusted p-values. Statistical significance level was considered at *p* < 0.05.

### Qualitative data collection and analysis

Qualitative data was collected through a series of FG including working equid owners and users, cart or harness makers and local veterinary professionals. As 80% of thematic content can be obtained from up to three FG [38] and data saturation can be reached by eight [39], we conducted three FG in each study location totalling nine overall. Each FG included between seven and ten participants [40]. Recruitment of FG participants was done through using an opportunistic approach to contact working equid owners and drivers in the selected regions, and through local cart-horse associations and equipment-repair workshops in the case of non-driver participants. A demographic survey was completed by all participants at the start of the session. Each FG consisted of a group discussion moderated by a researcher (BE) trained on qualitative data collection methods and proficient in both English and local language, and facilitated by another researcher and note-taker (AL) also trained on FG and a fluent speaker. Each FG included moderated group discussion and participatory exercises, exploring three key topics in the following order: (1) what constitutes an ideal cart and harness (including open discussion, a participatory ranking exercise of ideal work equipment attributes and a group drawing exercise of an ideal cart and harness); (2) what are the barriers and potential solutions to achieving the ideal cart and harness; and (3) what are the consequences of inadequate work equipment to equids and their users. In the first participatory exercise of identification and ranking of categories that define an ideal cart and harness, participants were asked to identify factors and attributes associated with what they considered to represent ideal work equipment. The FG moderator then grouped these attributes in consultation with participants, creating categories of equipment attributes by participant consensus. Participants were then asked to rank categories in order of importance, from the most to the least important attributes of ideal work equipment. For the drawing exercise, participants were split into sub-groups of 3–4 people and were given approximately 15 min to draw what they considered an ideal cart and harness over a pre-printed template of an equid silhouette. Each sub-group then presented and explained their drawings to the remaining participants. All sessions lasted a maximum of 90 min. Outputs of participatory ranking and drawing exercises were photographed, and discussions were recorded using a portable voice-recorder and subsequently transcribed and translated to English for analysis by BE.

Demographic surveys of FG participants were entered into REDCap [33, 34], and descriptive statistics generated using R software (2021 R Core Team [35]). Transcripts from FG were imported into NVivo 14 for Windows (QRS International, Australia) [41] for data management and analysis. Categories of attributes that constitute an ideal harness as identified by FG participants are presented, as well as the outcomes of the participatory ranking exercise. Rankings from individual FG were combined to present an overall ranking of harness attribute categories: the scoring position for each category was represented by a number equivalent to its rank within the total number of categories, and the sum of ranks for the same category across all FG was then subtracted from the total number of ranking exercises.

Thematic analysis [42] was performed on FG transcripts and participatory drawings to identify patterns of meaning within the data. After familiarisation with the transcripts, codes were created representing similarities and common patterns of data in the context of the research questions being investigated. Coded data were then organised into overarching themes that reflected connections or resemblances between codes. Similar codes and themes were reflected across FG without emergence of new perspectives towards the final sessions, suggesting thematic saturation had been reached. Quantitative and qualitative results were then integrated in a concurrent triangulation mix-methods approach [43–45] to cross-validate and interpret relationships between the data obtained. Where relevant, evidence from questionnaire responses was used to weight and understand the relative importance of different codes and to corroborate the validity of themes identified. Themes were then assessed in relation to each other as well as in relation to the central research questions and are presented in an overall thematic framework.

## Results

### Working equid surveys and cart-driver questionnaires (quantitative data)

A total of 369 working equids and their users were surveyed. One survey was incomplete and was removed from analysis resulting in a final study sample of 368 working equids and their cart-drivers in Bishoftu (32.9%; 121/368), Fiche (33.4%; 123/368) and Shashamene (33.7%; 124/368). Animals surveyed included horses (66%; 243/368), donkeys (33.2%; 122/368) and mules (0.8%; 3/368). The predominant work type performed by the equids was taxi work (median 60%; inter-quartile range [IQR] 0–80%), followed by transport of goods (median 25%; IQR 10–75%) and transport of water (median 0%; IQR 0–10%).

### Equipment characteristics

**Fig. 3:**
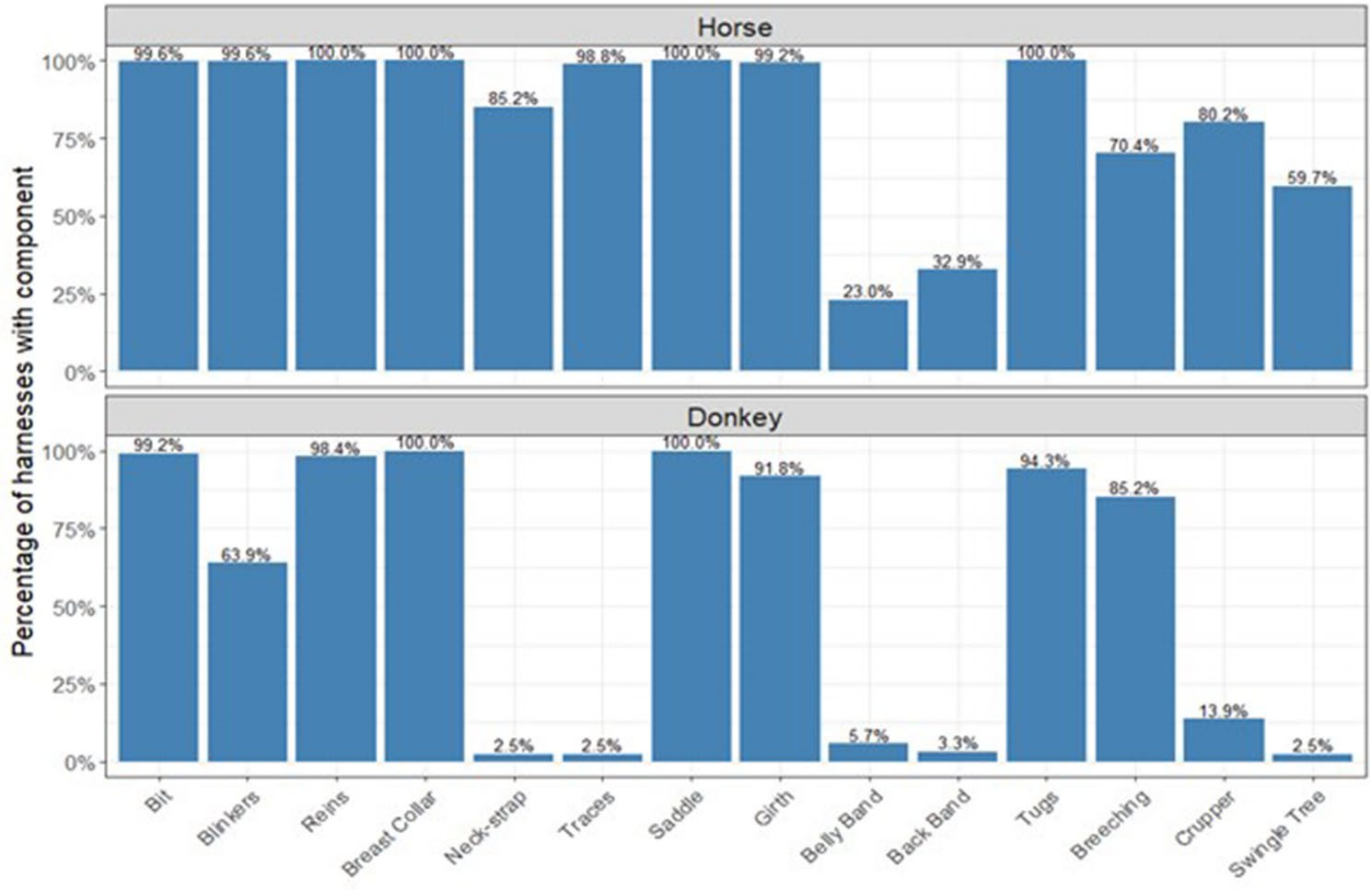
Proportion of harness components present in the equipment observed in a cross-sectional study of 368 working equids in three Ethiopian locations - Bishoftu, Fiche and Shashamene in 2022. The figure refers to the presence or absence of components in horses (top, *n* = 243) and donkeys (bottom, *n* = 122). The functionality of the different elements is not represented

Overall, none of the observed harnesses were considered fully adequate (0/368). All equipment sampled included a saddle and a breast collar (100%; 368/368) (Figs. 2 and 3). A swingle tree was present in 41% (151/368) of equids while a functional swingle tree was present in 19.6% (72/368). 13% (48/368) of equids had functional steering, functional breeching was present in 16.3% (60/368), functional saddle in 9% (33/368), functional weight distribution in 1.4% (5/368) and functional traction in 4.6% (17/368). In only 2.7% (10/368) of harnesses were all surfaces in contact with the animal (saddle padding, breast collar, neck-strap, girth, breeching and crupper) made from breathable materials. The proportion and frequency of sampled equipment components and characteristics are described in Supplement 2a. When stratified by species, donkeys had neck-straps, traces and swingle trees present in 2.5% (3/122), a back band present in 3.3% (4/122), and a belly band present in 5.7% (7/122). In horses, the least prevalent component was the belly band (23%; 56/243) followed by back band (32.9%; 80/243) and swingle tree (59.7%; 145/243).

Donkey harnesses were more likely to include breeching compared to horse harnesses (OR 2.4; 95% CI 1.4–4.3; *p* = 0.002) and to have saddles positioned at the base of the withers (OR 2.6; 95% CI 1.6–4.3; *p* < 0.001), while horse harnesses were more likely to include blinkers, girth, belly band, crupper, back-band, traces and neck straps compared to donkey harnesses (Table 1). Horse equipment was also more likely to include a functional swingle tree (OR 19.2; 95% CI 5.3–69.4; *p* < 0.001) and functional traction (OR 18.8; 95% CI 1.1–319.5; *p* = 0.04) compared to donkeys (Table 1).

**Table 1:** Significant species differences identified in univariable logistic regression for the presence and characteristics of harness components in a cross-sectional study investigating work equipment and practices of working equids in three Ethiopian locations - Bishoftu, Fiche and Shashamene in 2022. The proportion as well as the frequency (inside brackets) of each species with a given harness component is presented. Only horses were compared to donkeys for inter-species comparisons

Harness characteristics	Horses with component (*n* = 243)	Donkeys with component (*n* = 122)	Odds Ratio	95% CI	p-value	FDR adjusted p-value^†^
Blinkers	99.6% (242)	63.9% (78)	91.7	17.5–478.5	< 0.001	< 0.001
Girth	99.2% (241)	91.8% (112)	9.0	2.2–36.6	0.002	0.003
Belly band	23% (56)	5.7% (7)	4.9	2.2–11.2	< 0.001	< 0.001
Crupper	80.2% (195)	13.9% (17)	25.1	13.7–45.9	< 0.001	< 0.001
Back-band	32.9% (80)	3.3% (4)	13.0	4.9–34.6	< 0.001	< 0.001
Traces	98.8% (240)	2.5% (3)	2346	522–10,544	< 0.001	< 0.001
Neck strap	85.2% (207)	2.5% (3)	194	63.1–597	< 0.001	< 0.001
Swingle tree	59.7% (145)	2.5% (3)	58.7	18.1–190	< 0.001	< 0.001
Breeching	70.4% (171)	85.2% (104)	0.4	0.2–0.7	0.002	0.003
Appropriate positioning of saddle	60.1% (146)	79.5% (97)	0.4	0.2–0.6	< 0.001	< 0.001
Appropriate positioning of breast collar	18.5% (45)	8.2% (10)	2.5	1.2–5.2	0.012	0.016
Breathability of saddle padding materials	33.7% (82)	7.4% (9)	6.4	3.1–13.3	< 0.001	< 0.001
Breathability of collar materials	83.5% (203)	45.1% (55)	6.0	3.7–9.8	< 0.001	< 0.001
Breathability of girth materials	95.5% (232)	12.3% (15)	150.4	66.7–339	< 0.001	< 0.001
Breathability of crupper materials	46.5% (113)	3.3% (4)	22.9	8.6–61	< 0.001	< 0.001
Breathability of breeching materials	58.4% (142)	22.1% (27)	5.0	3.0–8.1	< 0.001	< 0.001
Presence of a functional swingle tree*^1^	28.4% (69)	1.6% (2)	19.2	5.3–69.4	< 0.001	< 0.001
Presence of a functional saddle*^2^	12.8% (31)	0% (0)	36.4	2.2–606.1	0.012	0.016
Presence of a functional traction system*^3^	7% (17)	0% (0)	18.8	1.1–319.5	0.042	0.048
Presence of functional breeching*^4^	22.6% (55)	3.3% (4)	7.8	2.9–20.9	< 0.001	< 0.001

*^1^ Swingle tree is present and moves freely. Traces attach to swingle tree

*^2^ Adequately positioned, wide pressure points, has gullet, has padding, secured tightly to the animal

*^3^ Adequate position over the point of draught, freely moving traces that attach to a functional swingle tree

*^4^ Presence of breeching and correctly assembled as either true or false breeching

^†^ False discovery rate correction (FDR) using the Benjamini-Hochberg method [37], adjusting for multiple comparisons

Harnesses from animals used predominantly for taxi work were more likely to have neck straps, back band, belly band, girth, crupper, blinkers, traces and functional swingle trees, saddles and breeching compared to other work types, while transport of goods was associated with having breeching and adequate saddle positioning (Supplement 2a). However, species and the predominant type of work were highly correlated. Horses were more likely to be doing predominantly taxi work (230/243; 94.7%) when compared to donkeys mostly transporting goods (100/122; 82%), water (10/122; 8.2%) or doing mixed work (10/122; 8.2%; *p* < 0.001).

### Cart-driver questionnaires

Median driver age was 28 years (range 24–70), and 98.9% (364/368) were male, with a median of three dependents (range 0–8) and an early secondary level of education (29.1%; 107/368) (Supplement 2b, Table 1). The median level of experience was 4 years (range 1–32), and most drivers derived 100% of their income from cart driving (range 40–100%) and considered their economic comfort level as ‘just managing’ (67.9%; 250/368). Most respondents (65.2%; 240/368) disliked cart-driving. Respondent demographics are shown in Supplement 2b and questionnaire responses regarding training, equipment and work practices are presented in Table 2.

Nearly all respondents that purchased their equipment did so from harness or cart makers (98.8%; 337/341). Of respondents that partially made their equipment at home, 35.5% (11/31) did so due to cost and 32.3% (10/31) due to availability. Types of equipment maintenance consisted of inspecting (73.9%; 272/368 for harnesses; 74.5%; 274/368 of carts), cleaning (38.9%; 143/368 for harnesses; 41.3%; 152/368 of carts), brushing (13.3%; 49/368 for harnesses; 29.1%; 107/368 of carts), oiling (3%; 11/368 for harnesses; 7.9%; 29/368 of carts) or other maintenance types (21.2%; 78/368 for harnesses; 21.7%; 80/368 of carts). The mean harness cost was ETB 4,213.60 (SD ± 3457.3) and mean cart cost was ETB 17,908.10 (SD ± 8147.3), approximately US$ 77 and US$ 325 respectively in November 2023.

**Table 2:** Responses from questionnaires to cart-drivers in a cross-sectional study of 368 working equids and their drivers across three Ethiopian locations - Bishoftu, Fiche and Shashamene in 2022. Results presented are stratified by horse and donkey drivers. Overall responses also include mule drivers (*n* = 3)

Questionnaire Responses	Horse drivers		Donkey drivers		Overall respondents	
	Frequency	%	Frequency	%	Frequency	%
**Source of training as a driver**						
Other Drivers	97/243	39.9%	83/122	68%	180/368	48.9%
Family	95/243	39.1%	39/122	32%	135/368	36.7%
No formal training	51/243	21%	0/122	0%	53/368	14.4%
**Source of training about harness assembly and hitching**						
Observation and intuition	184/243	75.7%	68/122	55.7%	255/368	69.3%
Other drivers	41/243	16.9%	48/122	39.3%	89/368	24.2%
Harness maker	18/243	7.4%	6/122	4.9%	24/368	6.5%
**Equipment source**						
Purchased	199/242	82.2%	110/121	90.9%	310/366	84.7%
Partly Purchased & Home-made	28/242	11.6%	1/121	0.8%	31/366	8.5%
Donated	5/242	2.1%	8/121	6.6%	13/366	3.5%
Inherited	10/242	4.1%	2/121	1.7%	12/366	3.3%
**Priority factor influencing decision when choosing work equipment**						
Cost	179/243	73.7%	67/122	54.9%	248/368	67.4%
Design	56/243	23%	48/122	39.3%	105/368	28.5%
Ease of Use	5/243	2.1%	4/122	3.3%	9/368	2.4%
Materials	1/243	0.4%	2/122	1.6%	3/368	0.8%
Popularity	0/243	0%	1/122	0.8%	1/368	0.3%
Maker	1/243	0.4%	0/122	0%	1/368	0.3%
Availability	1/243	0.4%	0/122	0%	1/368	0.3%
Equipment receives routine maintenance	204/243	84%	101/122	82.8%	308/368	83.7%
Driver is responsible for the choice of cart and harness	225/243	92.6%	106/122	86.9%	334/368	90.8%
**Person responsible for the equipment maintenance**						
Driver	125/203	61.6%	75/99	75.8%	202/305	66.2%
Designated maintenance person	74/203	36.4%	24/99	24.2%	99/305	32.5%
Family member	3/203	1.5%	0/99	0%	3/305	1%
Harness maker	1/203	0.5%	0/99	0%	1/305	0.3%
**Frequency of harness maintenance**						
Daily	86/243	35.4%	30/122	24.6%	117/368	31.8%
Weekly	55/243	22.6%	55/122	45.1%	110/368	29.9%
Monthly	49/243	20.2%	13/122	10.6%	63/368	17.1%
Yearly	13/243	5.3%	3/122	2.5%	16/368	4.3%
Other (as needed)	40/243	16.5%	21/122	17.2%	62/368	16.8%
**Frequency of cart maintenance**						
Daily	83/243	34.2%	12/122	9.8%	96/368	26.1%
Weekly	55/243	22.6%	34/122	27.9%	89/368	24.2%
Monthly	52/243	21.4%	51/122	41.8%	104/368	28.3%
Yearly	13/243	5.3%	4/122	3.3%	17/368	4.6%
Other (as needed)	40/243	16.5%	21/122	17.2%	62/368	16.8%
Driver is responsible for assembling and hitching the equipment	234/243	96.3%	120/122	98.4%	357/368	97%
Driver removes the harness during breaks	214/243	88.1%	122/122	100%	339/368	92.1%
Driver feels current equipment is comfortable for the animal	220/243	90.5%	119/122	97.5%	342/368	92.9%
Driver believes the harness influences animal’s ability to work	88/243	36.2%	62/122	50.8%	152/368	41.3%
Driver believes the cart influences animal’s ability to work	87/243	35.8%	60/122	49.2%	147/368	39.9%
Driver would like to change something about their equipment	110/243	45.3%	61/122	50%	171/368	46.5%
Driver believes the equipment is assembled and hitched correctly	223/243	91.8%	117/122	95.9%	343/368	93.2%
Driver feels current equipment is efficient	226/243	93%	118/122	96.7%	347/368	94.3%
Driver considers load distribution when loading the cart	214/243	88.1%	117/122	95.9%	334/368	90.8%

Significant associations were found between equipment source and harness characteristics, with equipment ‘Partly purchased and partly home-made’ being more likely to include various components such as crupper (OR 6.2; 95% CI 2–20; *p* = 0.002) or neck-strap (OR 2.62; 95% CI 1.12–6.25; *p* = 0.048) than equipment exclusively ‘Purchased’, and be made from breathable materials such as breathable girths (OR 4.31; 95% CI 1.37–14.29; *p* = 0.021) (Supplement 2b). There was no significant difference between the cost of ‘Partly purchased and partly home-made’ and ‘Purchased’ harnesses. The source of training on harnessing and equipment was also associated with the presence, breathability and functionality of equipment components, such as harnesses that included a crupper more likely to belong to drivers that learned from harness makers rather than from other drivers (OR 3.89; 95% CI 1.33–11.32; *p* = 0.032), or having functional breeching being associated with learning from other drivers when compared to learning by intuition and observation (OR 2.23; 95% CI 1.20–4.17; *p* = 0.004). Significant associations between questionnaire variables and equipment characteristics are summarized in supplementary material (Supplement 2b).

## Focus group discussions (qualitative data)

### Focus group participant characteristics

Nine FG were conducted between the three study locations and attended by a total of 87 participants. Overall, 77 participants (88.5%; 77/87) were both owners and drivers of a working equid, while 10.3% (9/87) were harness or cart makers and one (1.1%; 1/87) was a veterinary professional (Supplement 2c). All participants were male and 64.4% (56/87) were between 31 and 40 years of age with a median of 8 years (range 2–30 years) of experience with equids. For all participants, their occupation relating to the working equid was the primary occupation and the median percentage of income derived from equid-related occupation was 100% (range 80–100%) (Supplement 2c).

### Thematic analysis and concurrent triangulation

**Fig. 4:**
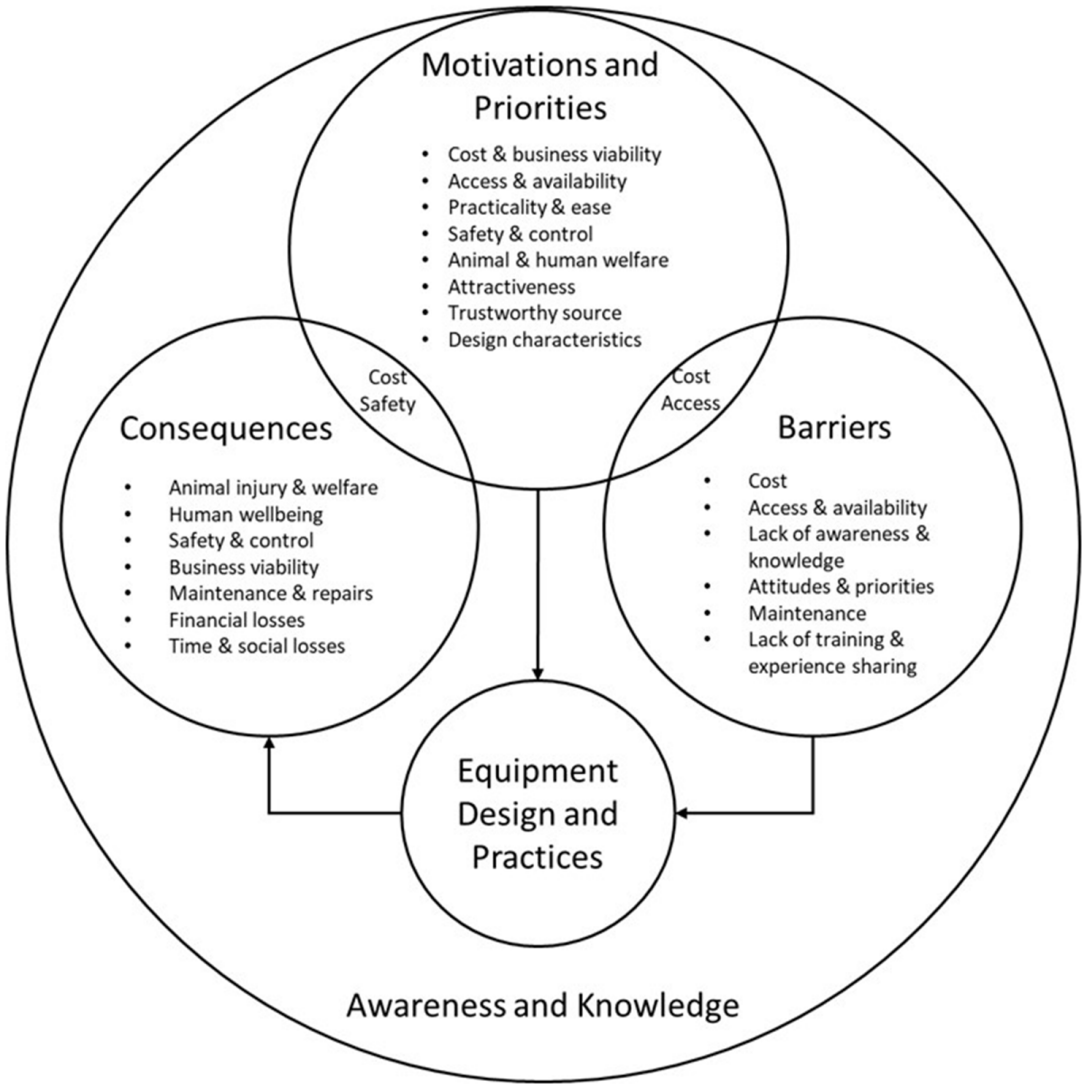
Thematic framework of overall factors involved in the use of working equid equipment, in a study investigating equipment design and practices in three Ethiopian locations - Bishoftu, Fiche and Shashamene in 2022. Key themes are identified and the main sub-themes contributing to each theme are listed. The relationship between themes and the central topic of equipment design and practices are illustrated with arrows, and where codes are common to different themes are represented as a circle overlap

A total of nine group ranking exercises of attributes of the ideal work equipment, as well as 20 drawings of equids hitched to what participants considered an ideal cart and harness were produced between all FG. Four main themes relating to the central topic of work equipment were identified through thematic analysis of FG transcripts and participatory exercises: (1) Motivations and priorities regarding equipment; (2) Awareness and knowledge of equipment design and function; (3) Barriers to using ideal equipment; and (4) Consequences of equipment design and practices. The thematic framework resulting from qualitative analysis is presented in Fig. 4 and illustrates the key elements contributing to work equipment design and practices as well as the relationship between them (Fig. 4). No new themes were identified in the last FG and it is therefore reasonable to believe that thematic saturation had been reached. Identified themes are described below with their respective supporting data.

#### Motivations and priorities regarding work equipment

Participants described a range of motivations that influenced the work equipment they used and looked for. Some factors were perceived as motivators only, while others were perceived both as motivators and barriers to ideal equipment (such as cost and access) or as both motivators and potential consequences of inadequate equipment they wished to avoid (such as cost and safety) (Fig. 4).

Several motivators were identified when discussing what would constitute the ideal work equipment. Participants raised both physical equipment attributes as well as factors relating to business viability and logistical qualities surrounding equipment use. Seven key attribute categories were identified by participants during the participatory exercises: ‘animal-friendly’, ‘appearance’, ‘availability’, ‘cost’, ‘design’, ‘practicality’ and ‘source’ (Table 3). Overall, participants prioritised ‘cost’ and ranked it as the most important category, followed by ‘animal-friendly’ and ‘design’.

I like the harness that is made by [the] harness maker around the market because it [does] not cause any wounds to my horse and he maintains my cart [at a] low price [Participant (P) BFG-12, horse owner and driver].

‘Appearance’ was ranked as the overall least important attribute of the ideal equipment (Table 3).

**Table 3:** Attributes characterising the ideal work equipment identified by 87 participants of nine focus groups in a study investigating work equipment design and practices in three Ethiopian locations during 2022 – Bishoftu, Fiche and Shashamene. The seven identified attribute categories identified during the participatory exercise are presented, together with examples of described attributes within each category and their overall ranking on participatory ranking exercises

Attributes	Example of described attributes	Overall position in participatory ranking
‘Cost’	Good value for money, affordable to purchase, repair and maintain.	**1**
‘Animal-friendly’	Prevents injury, comfortable, does no harm, enables free movement.	**= 2**
‘Design’	Strong, durable equipment with quality materials and the right shape.	**= 2**
‘Practicality’	Easy to use, lightweight, simple to assemble, easy to maintain.	**3**
‘Availability’	Easy to find, locally available equipment, locally maintained.	**4**
‘Source’	By trained professionals, from a popular, trusted, or credible source.	**5**
‘Appearance’	Beautiful, modern, attractive, has the right colours.	**6**

Quantitative results from cart-drivers questionnaire support the influence of cost as one of the main motivators, with over 67% of respondents considering the cost of equipment their priority when choosing work equipment (Table 2).

In addition to the ideal equipment attributes identified through the dedicated participatory ranking exercise, two further key motivators were identified thematically during group discussions: ‘human comfort and wellbeing’, and ‘safety and control’. Participants raised that equipment should not only enable efficient draught but also allow for efficient control of the animal to prevent accidents or injury to the equid, driver or customers. These overlapped with the potential consequences of inadequate equipment which participants were motivated to avoid and prevent.

[The] driver and customer seats must be comfortable [and] also safe for the horse and people [P SFG-16, horse owner and driver].

[The cart] must contain fully inflated [tyres], balanced wheels, [and the] driver and passenger seats [must have a] head-cover umbrella that protects from rain and sunlight [and] also increases its beauty. [P QJM].

#### Awareness and knowledge of equipment design and function

Technical knowledge of equipment design, assembly and hitching, as well as participant awareness of this knowledge and of the influence of design on work efficiency and wellbeing was another key theme identified.

Overall, participants demonstrated some awareness of the importance of equipment design, ranking it highly in the participatory exercises (Table 3). Existing technical knowledge included the requirement for several harness components, the importance of adequate cart balance, tyre inflation, the need for periodic maintenance and the need to use designs that cause no harm to the animal and protect its ability to work efficiently. The nature of harness materials, such as non-abrasive or breathable materials, was also among the most referenced equipment characteristics, and participants understood that design was important and that certain materials were better for their animals than others.

The ideal harness means [a] breast harness made from fabric [and] not from old tyres, [which] is good for the donkey, [and with] two fully inflated tyres [P SH-28, donkey owner and driver].

Most ideal harness categories can be bits, breast band, girth and crupper, [which] must be with the correct shape. [P SH-9, donkey owner and driver].

**Fig. 5:**
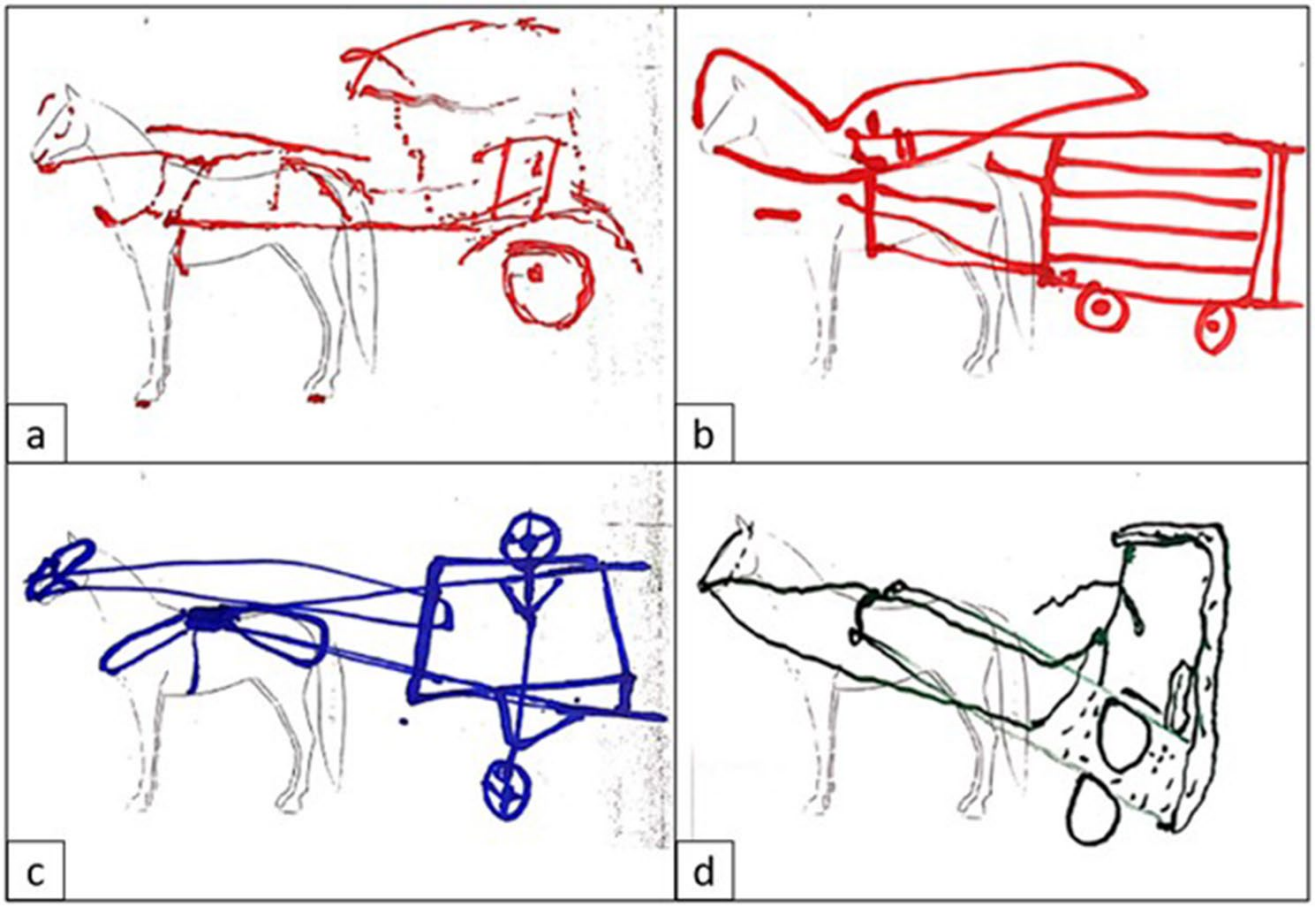
Example drawings from participatory exercises of nine focus groups in a study investigating working equid equipment and practices in three Ethiopian locations - Bishoftu, Fiche and Shashamene in 2022. Participants (*n* = 87) were asked to draw an ideal work equipment hitched to a working equid. The drawings illustrate the theme of awareness and knowledge of equipment design and function. Drawings depict gaps in the understanding of efficient traction dynamics, as shown in drawing **(c)** where the breast-collar is attaching to the saddle without the use of traces, or in **(d)** where both a breast-collar and traces are absent. Poor cart balance is illustrated in drawing **(d)**, where the shafts pass through the centre of gravity at an accentuated angle, as well as in **(a)** where in order for the shafts to be horizontal using a similar cart design the size scale between cart and animal is distorted. Additionally, with the exception of **(b)**, two-wheeled carts are the predominant choice of design. Gaps in the understanding of hitching and fitting of different harness components are illustrated in the ambiguity between the function of certain components (such as unclarity between what is a breeching or crupper in **(b)** and **(c)**, and complete absence of various components such as crupper **(d)**, traces **(c, d)**, breeching **(a, d)**, breast-collar or girth **(d)**

However, when prompted to draw the equipment or clarify which components were essential, a lack of knowledge regarding the recommended components for a harness was identified, in particular relating to elements that enable efficient traction such as traces and swingle trees (Fig. 5).

Additionally, the function and interaction of equipment components was seldomly raised. Participants were aware that equipment design can impact on the animal and driver, but few offered insight into why a given design might lead to the associated consequences, and showed little awareness of the role that hitching and driving practices may play towards these consequences. Several participatory drawings also reflected incorrect assembly and hitching of the equipment (such as attaching the collar or breeching to the saddle instead of to the traces or shafts respectively). No thematic differences were identified between drawings produced by groups integrating a harness-maker and those drawn by groups made exclusively of cart drivers. Figure 5 illustrates examples of FG drawings (Fig. 5).

Quantitative data support the theme of awareness and knowledge, where almost 70% of questionnaire respondents had learnt about harnessing and hitching through intuition and observation (Table 2). While over 90% of cart-drivers believed their equipment was efficient, hitched correctly and comfortable for the animal (Table 2), there was a clear mismatch between this belief and the observations of the surveyed equipment itself, where no harnesses were considered fully adequate. Furthermore, only around 40% of surveyed participants believed the cart or harness could influence the animal’s ability to work, supporting the lack of awareness and technical knowledge identified in thematic analysis (Tables 1 and 2; Fig. 5).

Knowledge and awareness of design were also identified in the context of other themes. The lack of awareness and knowledge represented a barrier for the use of ideal equipment, and was frequently identified in the context of motivations and potential solutions, where participants raised the need for training owners, cart-drivers and equipment-makers, as well as the need for raising awareness, acquiring equipment made by trained professionals and experience sharing among community members.

The main solution is having knowledge [about] the maintenance and cleaning of the cart and harness [P WND].

[We should] create awareness to others because most of the drivers use inappropriate harness materials due to ignorance or not giving much attention to the horse, even [if the] harness causes wounds [P UGN].

#### Barriers to using ideal work equipment

Participants raised several barriers to the use of the ideal work equipment, of which ‘cost’ was a key barrier identified in all FG. Both financial and time costs associated with the purchase, repair and maintenance of equipment were viewed as significant challenges.

There are so many influencing factors, like the cost of the harness materials in the market and shops are very expensive [while] the daily income generated from this activity is low. [P BFG-6, horse owner and driver].

Every cart and harness has its own rank which depends on the economic level of the cart owner or driver. For example, some people work with [a] low grade cart and harness, [while] some of them work with high grade or high standard cart and harness which [are] well-designed. [P GTS].

Additionally, participants linked lower-cost equipment with overall poorer equipment quality and design, which could in turn carry consequences to the animal.

But the main issue is the cost […]. If [a] harness is obtained at low price, every user can easily purchase [it] at low price. […]. This leads [to the] use of low-quality cart and harness which can affect the horse performance and its health. Harnesses with low quality do not fit the animal due to improper design, that causes wounds [on the] animal because the harness is not comfortable and [does] not fit the horse. [P BFG-17, harness maker].

Access and availability to affordable quality equipment and maintenance services were also viewed by participants as important barriers to adequate equipment design and use. This enhanced participants’ motivation to obtain equipment from trusted and quality sources.

There is no access to modern [and] new harness materials in this town; we only make harnesses from old tyre rubber and also we use nails to fix them [P BFG-1, horse owner and driver].

Previously when I purchased bits from [the] harness maker or market which [were] not suitable to my horse which caused wounds [and] even cut the tongue of my horse.” [P BFG-20, horse owner and driver].

This point generated mixed opinions in which certain groups argued that it is possible to produce quality equipment at home and identified home production of equipment as a potential solution to this challenge.

It is possible to make [an] ideal harness even [at] home or partly at home by purchasing the harness [materials] from the market. For example, sometimes the [available] harness is partially fulfilled and partially not fulfilled, it is possible to make [it into an] ideal harness at home also, but [this] may need money. [P GTS].

This proposed solution aligns with quantitative data findings where, although representing only 8.5% of questionnaire respondents, equipment that was partly purchased and partly home-made was more likely to include more of recommended harness components than exclusively purchased equipment (Supplement 2).

Other barriers identified by participants related to the knowledge, attitudes and practices of cart-drivers towards work equipment. These included poor maintenance practices, cost-driven attitudes with prioritization of low upfront costs, and low attention to animal and equipment care. Additionally, a lack of awareness and knowledge about equipment design, function and use was identified by participants, as well as a lack of experience sharing among the community. To address some of these challenges, participants identified a need for equipment-specific training and awareness across various sectors of the community, raising it as a potential solution. A change in attitudes and practices regarding cart-driving, equipment and animal care was also considered essential by participants.

The main problem [relating to] not using [an] ideal harness is not giving serious attention [to it]. Mainly, we don’t give full commitment when we select harness materials. This differs from person to person [and whether they] give care to [their] horse. [P BFG-3, harness maker].

#### Consequences of work equipment design and practices

Participants identified several potential consequences of using inadequate work equipment, impacting both animals and people. The main consequences to animals related to health and performance implications, either directly as equipment-related wounds, discomfort or restricted movement or breathing; or indirectly as secondary health implications of equipment-related wounds such as inability to eat or drink, contracting secondary conditions and potentially death.

[If the equipment is not adequate it] causes injury [and] wounds on the horse and [it] stops working efficiently; and [the] development of wounds exposes the horse to another disease like epizootic lymphangitis. [P BFG-6, horse owner and driver].

[Inadequate equipment] also decreases working efficiency and [leads to] loss [of] body condition. [P BFG-3, harness maker].

Several ways in which inadequate equipment can affect people were raised by participants, ranging from comfort and user experience implications to impacts on lifestyle, and income, time and asset losses. Participants also demonstrated concern about human health, wellbeing and business viability as consequences of inadequate equipment.

Also, if the horse is wounded very seriously [because of inadequate equipment] there is [a] bad smell that may affect the health of driver, [and then] users and the passengers do not prefer to use transportation by this horse [P BFG4, horse driver].

Safety concerns were raised in various discussions, where participants identified that inadequate equipment compromised effective control of the animal and vehicle, increasing the risk of accidents and injury.

When [the] harness is not adequate [it is] difficult to control [the] donkey and [that can lead to] collisions with [a] car or any other object that cause damage to the driver, people around the cart and [the] donkey [P MKT].

Several economic implications of inadequate equipment were identified by participants. Participants considered cost a major limitation to providing and maintaining quality equipment. Inadequate equipment was in turn considered to have further economic consequences due to the associated need for additional repairs, veterinary costs that may include loss of an animal and reduced potential for deriving income. This in turn was described as having severe financial impacts on the entire household.

[When equipment is not adequate it] causes economic crisis to the family. Because [it] decreases [the] daily income obtained from [the] cart occupation [P BFG2, horse owner and driver].

There is also [the] treatment cost for the horse that [has injuries] caused by inadequate equipment [P BFG4, horse owner and driver].

The whole family that depends on the income can be affected [P BFG7, horse owner and driver].

Additionally, social, emotional and time costs were identified, such as those associated with the requirements to care for an injured animal or replacing the animal in its role.

If the equipment is not adequate, donkey and driver [are] affected because [the] donkey [does] not work properly. […] At that time [the] driver [needs to] work equally with the donkey to support him by pulling the cart so [the] driver works like [a] donkey, the driver becomes the second donkey unless the donkey works efficiently. [P MKT].

## Discussion

This study aimed to investigate the equipment used by working equids in three Ethiopian locations, and understand the knowledge, attitudes and practices of stakeholders regarding work equipment. In this study, equipment characteristics differed considerably from the recommended harnessing principles and guidelines [11, 13, 16, 18], and donkeys used an overall inferior equipment to that of horses. When selecting work equipment, cart-drivers were motivated by several factors, of which cost was considered a priority. Inadequate equipment was found to have several implications to both animals and people, and various barriers to obtaining the ideal equipment were identified, centring around a direct or indirect lack of access.

Equipment observed in this study deviated significantly from the available recommendations and guidelines for breast-collar harnesses [11, 13, 16, 18], with none of the harnesses being considered fully adequate due to several design and hitching flaws. Previous studies suggest that inadequate work equipment is common among working equids in Ethiopia [6, 7, 46], with less than a third of donkeys in the Dale district using what was considered improved harnesses [47], although a detailed description of what components were included are missing in this study as well as what the improvements related to. Further, median quality scores for mule harnesses were reported as 3.5/5 in the Amhara region [6]. This has been linked to health outcomes such as lameness [6] and harness-related wounds [6, 22, 24], and attributed as the cause of reduced work outputs in up to 76% of working donkeys in other African countries [48]. However, functionality of equipment was not described in available reports and insight into what makes the equipment inadequate is scarce, limiting strategies towards improvement. This study suggests that belly-bands, back-bands and swingle trees are the most notably missing elements, with most donkey harnesses also lacking traces and a neck-strap. Even if present, the functionality of those elements as a whole was often low due to design, assembly and hitching weaknesses that meant certain components were not fulfilling their intended function. For example, even if traces were present and attached to a swingle tree, they were frequently wrapped around the shafts preventing their free movement and therefore hindering the purpose of the swingle tree. Mapping of specific equipment inadequacies, such as those concerning traction and weight distribution, may enable small modifications to the existing equipment such as the use of traces or position of the shafts that may consequently contribute to an improvement in traction efficiency and animal welfare [9, 11, 13, 15, 49, 50]. Moreover, weaknesses identified include not only the equipment itself but also the way in which equipment is used and assembled. While several of the recommended components were missing from the observed harnesses, there was at the same time a wide prevalence of components not considered essential and which can represent a welfare risk. For example, the majority of equids and particularly of horses observed were wearing blinkers. Blinkers are considered an optional part of the equipment [13, 18] and, while advantageous in some cases, often lead to negative welfare impacts such as ocular trauma and injury [51–53] as well as increased stress markers in face of unfamiliar sounds and environments [54]. The prioritisation of blinkers in relation to other harness components may reflect ingrained cultural practices and beliefs and provides an example of where design and investment could be redirected towards more functional overall equipment through awareness and education. As such, in addition to design recommendations, strengthening equipment-related knowledge and behaviour change towards improved practices is needed and could lead to higher equipment functionality and efficiency [49, 50].

Marked species and work-type differences in the design and functionality of equipment were observed, with donkey harnesses being of generally lower standard. Other studies suggest donkeys often have poorer welfare indicators [55, 56] and are perceived as less valuable [5, 57] than other equid species, which could contribute to these findings. Horses in this study performed predominantly taxi work, where the equipment is immediately relevant to the income-generating activity and may therefore be of a higher standard. Additionally, the role and welfare of equids can vary significantly within Ethiopia [58] and one of the study locations (Shashamene) contributed to almost the entirety of donkeys surveyed, so species variation may be explained by local community context. Nevertheless, this suggests equipment concerns differ across populations and species of animals and highlights the need for appropriate and context-relevant interventions for each group.

Several motivators were found to influence the choice of work equipment, with affordability of equipment being considered a priority. This is perhaps unsurprising in a LMIC context where working equid users face significant economic pressures [5, 59, 60]. In line with this motivation, unaffordability was perceived as one of the main barriers to quality equipment. The importance of cost was evident not only in terms of the upfront value of equipment, but also in relation to the supporting services for equipment upkeep and repair. Financial, time and social costs involved with the maintenance of equipment, treatment of equipment-related injuries, ability to retain customers or conduct income-generating activities were found to be significant impacts of inadequate equipment use, suggesting the cost of the equipment itself is only one part of the problem. As such, cost is both one of the main motivators and barriers to using adequate work equipment, as well as an important consequence of not using it. Furthermore, less than one per cent of respondents said they enjoyed cart-driving, which may be linked to the low attention to animal and equipment care raised as a barrier in FG discussions. However, other studies have shown that in some contexts the animal welfare index is not associated with socioeconomic status and access to resources [61], and that welfare improvement may also be achieved through empathetic attitudes and motivations. While complex economic influences cannot be overlooked, improving the core design of existing equipment through training and awareness, or complementing it through partial home-production as identified by our study participants as a potential solution, may help improve overall equipment quality in an affordable way and reduce the likelihood of cost-related consequences.

A limited technical knowledge of recommended equipment design, hitching and draught dynamics was identified in both survey and FG data. Most cart-drivers had no formal training in regard to equipment, and while those learning from other drivers or harness makers were more likely to have certain functional elements, drivers who learned by intuition and observation alone often used more complete and breathable equipment, suggesting a knowledge gap across groups and training sources. Additionally, most FG harness-makers had no formal training in harness making. This knowledge gap contributes to the lack of access to quality equipment and distrust in available sources. Although the importance of design was ranked highly by FG participants, this may have been influenced by bringing attention to the topic during the group discussions and hence raising awareness where it may not normally be present. Furthermore, although not included in questionnaires, harness makers took part in FG and may be predisposed to considering design due to the nature of their work, therefore likely adding to the awareness observed in the groups. Still, the need for training and awareness was identified by participants as a barrier and potential solution. Although it has been shown that education level of working equid owners does not necessarily correlate with their animal’s welfare [61], the use of work equipment requires specific technical knowledge and understanding that is not provided through conventional schooling. Additionally, equipment design and its ability to protect animal welfare were considered important motivators when selecting equipment, and almost half of questionnaire respondents would indeed like to change something about their equipment suggesting an openness to modifications. This suggests that although the design is not always appropriate, which is known to impact on animal welfare [6, 22, 24, 27], participants in this study were already motivated towards those attributes and have a favourable attitude. This represents an encouraging opportunity to complement this gap with adequate training and capacitation of cart-drivers as well as cart and harness-makers.

Beyond the financial accessibility, an actual lack of access to quality equipment was identified in the FG as a key barrier. Supplier credibility and reputation, as well as the quality of product offered, were valued by participants but considered hard to find. Associations were also found between equipment source and the presence of key components in surveyed animals, where partially home-made equipment was often more functional than those exclusively purchased. Although cost remains a central limitation, no significant difference was found between the cost of purchased and partly home-made equipment. Other authors suggest that affordable home-made components, such as collars, can be fit for purpose [12], and partial home-production by trained community members has also been identified by FG participants as a potential solution. Therefore, empowering equid owners and users to fill the remaining needs gap with the production or adjustment of equipment components in an educated way could contribute to sustainably improving access to functional equipment.

Participants of this study identified several consequences of inadequate work. Direct injury to the animal is a key concern, which aligns with findings from several authors reporting high prevalence of harness-related wounds in Ethiopia [22, 25, 26]. Additionally, inadequate equipment was found to have several direct and indirect consequences for people. These include impacts on human comfort and wellbeing, accidental injury and secondary implications of lower equipment and animal efficiency. Road traffic accidents associated with a reduced control over the animal when equipment is unfit was an important consequence identified and cart-drivers were motivated by safety. Given the high frequency of traffic accidents in working equids [59, 62–64], this represents an important public health concern and optimising equipment design may help decrease the prevalence of accidents and injury. The use of inadequate equipment also hinders the income-generating capacity and carries additional financial implications, related not only to higher need for repairs and replacement, but also animal treatment costs, reduced draught efficiency [49] and animal productivity, as well as the associated time costs. Due to the role working equids play in their communities, the impact of inappropriate equipment on the ability to generate a livelihood often extends to the entire household [5, 65] and must therefore be viewed within a One Health [66] and One Welfare [67] framework.

The fact that work equipment was not assessed in motion and no indicators of draught kinematics were measured are a limitation of this study and interpretations of equipment efficiency are in part based on theoretical assumptions. However, this observational study describes and measures indicators according to recommended harness designs [8, 9, 13, 15]. Classification of certain equipment attributes, such as cleanliness, have a degree of subjectivity and could be influenced by environmental conditions or how long the animal has been at work. Nonetheless, all surveys were conducted by the same two trained assessors to help standardise assessments. Some equipment differences between species have wide confidence intervals which must be interpreted cautiously and further investigated. However, despite the high variation in the data, statistical adjustments for multiple comparisons had minimal impact on the associations found. Only male cart-drivers were included in the FG and there was an overall low percentage of female cart drivers (1.1%) in the cross-sectional survey, while it is often women who take care of working equids [68, 69] and possibly of the maintenance of their equipment as identified through the driver questionnaires. Female perspectives on equipment in the qualitative data are therefore underrepresented and should be further investigated. Finally, findings from this study are constrained to sampled communities with regional associations and caution is needed in extrapolating results to other contexts and work types.

### Conclusions and recommendations

Significant weaknesses with the design and use of working equid equipment were identified in this study that carry consequences to the welfare of working equids as well as the broader community. To improve this, enhancing access to quality and affordable equipment designs is paramount, as is a shift in human behaviour towards more efficient equipment use and practices. A number of harnessing guidelines and training resources have been developed [10, 11, 36] and targeted at working equid users. Yet, inadequate equipment design and use remain common [6, 7, 19, 46] and a low user awareness of how this relates to equid welfare and efficiency was found. This study describes specific flaws in the equipment, and we hypothesize that problem-specific solutions may increase the uptake of given recommendations. As such, we propose that training initiatives should be aimed at both equid users and equipment manufacturers, with an initial focus on the foundational knowledge of basic equipment components, their purpose and functionality. A second tier of training would then consolidate foundational knowledge and should be tailored to the relevant group: focusing on manufacturing and enhancing equipment design and quality for cart and harness-makers based on the identified weaknesses; and addressing fit, hitching and maintenance considerations for working equid users. Cart-drivers from this study were already motivated towards equipment design and animal welfare. Promoting awareness, technical training and support of manufacturers and community-based activities could therefore result in a more sustainable improvement in the quality of work equipment and practices, ultimately leading to One Health and One Welfare benefits.

### Electronic supplementary material

Below is the link to the electronic supplementary material.

Supplementary Material 1

Supplementary Material 2

## Data Availability

Data is provided within the manuscript or supplementary information files. Datasets used during the current study are available from the corresponding author upon reasonable request.
