# Supplementary material for: Putting the cart before the horse: mixed-methods participatory investigation of working equid harnessing practices in three selected towns of the Oromia national regional state in Ethiopia
Source: BMC Vet Res. 2024 Mar 22;20:113. doi: 10.1186/s12917-024-03967-3 (PMC10958837; doi:10.1186/s12917-024-03967-3)
Supplement: Supplementary file 1 — Supplementary Material 1 [file 12917_2024_3967_MOESM1_ESM.pdf]

Supplement 1 – Data collection sheets for the survey of animal welfare and work equipment indicators and questionnaires to the respective cart-driver

Animal-cart ID ☐

Animal-Cart Unit Record

Town: ☐ Bishoftu ☐ Shashamene ☐ Selale

Location: ____________________

Date: ___/___/____

Animal Signalment

Species: ☐ Donkey ☐ Horse ☐ Mule

Gender: ☐ Female ☐ Male Entire ☐ Male Castrated

Age: _______ *years*

Height: _______ *cm*

Breed: ____________________

Work

Work Type:
____% Taxi (people)
____% Transport goods
____% Transport water
____% Other: ______________

Work per week: ______ *days*

Work per day: ______ *hours*

Average planned breaks during work: _______ *breaks*

Average duration of each planned break: ______ *minutes*

Welfare Parameter Scores

General Attitude: ☐ Positive ☐ Non-reactive ☐ Negative

BCS: ☐ 1 - Very thin ☐ 2 - Thin ☐ 3 - Ideal ☐ 4 - Fat ☐ 5 - Very fat

Gait: ☐ Not lame ☐ Moderately lame ☐ Severely lame ☐ Not weight-bearing ☐ Not assessed

Response to Spinal Contact: ☐ No reaction ☐ Weak reaction ☐ Strong reaction

Limb Score:

Front	Hind
☐ 0	☐ 0 *(normal)*
☐ 1	☐ 1 *(1 abnormal)*
☐ 2	☐ 2 *(both abnormal)*

Hoof Score:

Front	Hind
☐ 0	☐ 0 *(normal)*
☐ 1	☐ 1 *(1 abnormal)*
☐ 2	☐ 2 *(both abnormal)*

Shoes: ☐ x 1 ☐ x 2 ☐ x 3 ☐ x 4 ☐ No Shoes

Shoe Type: ☐ Metal ☐ Rubber ☐ Other: ________

Harness Removed for Assessment: ☐ BCS ☐ Gait ☐ Spinal Contact ☐ Wounds

Wounds

☐ *Photos*

Size: 1 = small; 2 = medium; 3 = large / Severity: 1 = superficial/healed; 2 = skin and subcutaneous; 3 = deeper structures

Harness-Related Wounds

	Number	Size (1-3)	Severity (1-3)
Bit			
Blinker			
Chest-band			
Girth			
Saddle			
Tail			
Shafts			

Other Wounds

	Number	Size (1-3)	Severity (1-3)
Encouragement			
Hobble			
Traditional Practices			
Limbs			
Other			

Harness

Transmission

Breast collar ☐ *Photo*

- Present: ☐ Yes ☐ No
- Width: _____ *cm*
- Material: ☐ Fabric ☐ Nylon ☐ Leather ☐ Rubber ☐ Rope ☐ Other: _____
- Position: ☐ Pectoral ☐ High ☐ Low
- Adjustable: ☐ Yes ☐ No
- Padding: ☐ Yes ☐ No
- Thickness: ☐ Adequate ☐ Inadequate
- Material: ☐ Fabric ☐ Nylon ☐ Leather ☐ Rubber ☐ Rope ☐ Other: _____

Neck Strap ☐ *Photo*

- Present: ☐ Yes ☐ No
- Adjustable: ☐ Yes ☐ No
- Material: ☐ Fabric ☐ Nylon ☐ Leather ☐ Rubber ☐ Rope ☐ Other: _____

Traces ☐ *Photo*

- Present: ☐ Yes ☐ No
- Movement: ☐ Free ☐ Not Free
- Material: ☐ Fabric ☐ Nylon ☐ Leather ☐ Rubber ☐ Rope ☐ Other: _____

Swingle ☐ *Photo*

- Present: ☐ Yes ☐ No
- Traces attach: ☐ Yes ☐ No
- Moves: ☐ Yes ☐ No

Is the breast collar positioned through the point of draught? ☐ Yes ☐ No

Breeching ☐ *Photo*

- Present: ☐ No ☐ Yes - True ☐ Yes - False
- Assembly: ☐ Adequate ☐ Inadequate
- Material: ☐ Fabric ☐ Nylon ☐ Leather ☐ Rubber ☐ Rope ☐ Other: _____

Steering

Bit ☐ *Photo*

- Bit: ☐ Yes ☐ No
- Condition: ☐ Good ☐ Poor
- Size: ☐ Fit ☐ Not fit

Blinkers ☐ *Photo*

- ☐ Yes ☐ No
- ☐ Dished ☐ Flat
- ☐ Touching ☐ Not Touching

Reins ☐ *Photo*

- ☐ Yes ☐ No
- ☐ Move Freely ☐ Don't move freely

Weight Distribution

Saddle

- Present: ☐ Yes ☐ No
- Material: ☐ Wood ☐ Metal ☐ Other: _____
- Shape: ☐ Gullet ☐ No Gullet
- Position: ☐ Adequate ☐ Withers ☐ Back
- Fit: ☐ Tight ☐ Loose
- Pressure Points: ☐ Narrow ☐ Wide

Saddle Padding

- Padding: ☐ Yes ☐ No
- Thickness: ☐ Adequate ☐ Insufficient ☐ Excessive
- Description: _____

Back Band	Girth	Belly Band	Crupper	Tugs
☐ None	☐ None	☐ None	☐ None	☐ None
☐ Fabric	☐ Fabric	☐ Fabric	☐ Fabric	☐ Yes - loose
☐ Nylon	☐ Nylon	☐ Nylon	☐ Nylon	☐ Yes - tight
☐ Leather	☐ Leather	☐ Leather	☐ Leather	
☐ Rubber	☐ Rubber	☐ Rubber	☐ Rubber	
☐ Rope	☐ Rope	☐ Rope	☐ Rope	
☐ Other: _____	☐ Other: _____	☐ Other: _____	☐ Other: _____	

Photos: ☐ *Saddle* ☐ *Girth* ☐ *Backband* ☐ *Crupper* ☐ *Belly Band* ☐ *Tugs*

Do shafts pass through center of gravity?
☐ No ☐ Yes - Horizontally ☐ Yes - Not Horizontal

Appropriate shaft width?
☐ Yes ☐ No – Too wide ☐ No – Too narrow

Cart

- Number of animals: _____
- Number of wheels: _____
- Wheel type: ☐ Wood ☐ Pneumatic - good inflation ☐ Pneumatic - poor inflation
- Axle balance: ☐ Yes ☐ No

Is the girth adjustable? ☐ Yes ☐ No

Is the harnes clean? ☐ Yes ☐ No

Comments

Animal-cart ID ☐

Cart-Driver Questionnaire

Animal-Cart Unit Record

Town: ☐ Bishoftu ☐ Shashamene ☐ Selale

Location: ....

Date: __/__/____

Demographics

Gender: ☐ Male ☐ Female ☐ Other ☐ Rather not say

Age: ....
☐ Rather not say

Number of dependents: ....
☐ Rather not say

Level of Education: ☐ No formal education ☐ Primary education ☐ Early secondary ☐ Late secondary ☐ Tertiary education ☐ Rather not say

Occupation Relating to Working Equids

Ownership of assessed animal: ☐ Owner and driver ☐ Driver only ☐ Rather not say

Number of equids owned: ....

Experience as driver: .... *years*

Experience with equids: .... *years*

Is driving the main occupation: ☐ Yes ☐ No

Given your current needs and financial responsibilities, would you say that you and your household are: ☐ Finding it difficult ☐ Just managing ☐ Comfortable ☐ Very comfortable ☐ Prospering ☐ Rather not say

Approximate percentage of income derived from cart-driving? ....%

Enjoyment of driving occupation: *1=strongly dislike 5=strongly enjoy* ☐ 1 ☐ 2 ☐ 3 ☐ 4 ☐ 5

Source of Training as a Driver: ....

Choice of Equipment

Are you responsible for the choice of cart and harness for your animal? ☐ Yes ☐ No

If "No", who is? ....

Where have you obtained your harness equipment? ☐ Purchased ☐ Inherited ☐ Donated ☐ Home-made ☐ Partly purchased and home-made ☐ Other: ....

If "Home-made" or "Partly purchased and home-made", please explain why: ☐ Cost ☐ Availability ☐ Tradition ☐ Design ☐ Responsibility ☐ Other: ....

If "Purchased" or "Partly purchased", where was it purchased from? ....

What is the near cost of your harness? ....

What is the near cost of your cart? ....

Which factors influence your decision when choosing this equipment? *(select as many as apply)* ☐ Cost ☐ Design ☐ Materials ☐ Tradition ☐ Ease of use ☐ Popularity ☐ Maker ☐ Culture ☐ Location ☐ Recommendation ☐ Availability ☐ Other: ....

Which of these factors has the highest importance to you? *(select only one)* ☐ Cost ☐ Design ☐ Materials ☐ Tradition ☐ Ease of use ☐ Popularity ☐ Maker ☐ Culture ☐ Location ☐ Recommendation ☐ Availability ☐ Other: ....

Equipment Maintenance

Does the work equipment you use receive any routine maintenance?	☐ Yes	☐ No	☐ Not sure
Are you responsible for the routine maintenance of the equipment?	☐ Yes	☐ No	If "No", who is? ………………………

With what frequency does the work equipment you use receive maintenance and how is the maintenance best described?

Harness	Cart		Harness	Cart	
☐	☐	Daily	☐	☐	Inspecting
☐	☐	Weekly	☐	☐	Cleaning
☐	☐	Monthly	☐	☐	Brushing
☐	☐	Yearly	☐	☐	Oiling
☐	☐	Other: ……………	☐	☐	Other: ……………

Assembly and Hitching

Are you responsible for assembling the harness and hitching your animal?	☐ Yes<br>☐ No	If "No", who is?<br>………………………………………………	
How have you learnt about harness assembly and hitching?	☐ Intuition<br>☐ Observation	☐ Other drivers<br>☐ Harness maker	☐ Research<br>☐ Other: …………
Where do you go to for additional information about work equipment?	☐ Other drivers<br>☐ Harness maker<br>☐ Research	☐ Community<br>☐ Veterinary professionals	☐ NGOs<br>☐ Other: ………………
If possible, would you change anything about your current harness or cart?	☐ Yes ☐ Not sure<br>☐ No	If "Yes", what?<br>………………………………………………	
Do you believe your harness is currently assembled correctly?	☐ Yes	☐ No	☐ Not sure
Do you feel your current equipment is efficient?	☐ Yes	☐ No	☐ Not sure
Do you think about load distribution when loading the cart?	☐ Yes	☐ No	☐ Not sure
If "Yes", how do you aim to distribute the load?	………………………………………………		

Animal Equipment Interaction

Do you remove the harness from the animal during rest periods at work?	☐ Yes	☐ No	☐ Sometimes
Do you think the harness can influence the animal's ability to work?	☐ Yes	☐ No	☐ Not sure
Do you think the cart can influence the animal's ability to work?	☐ Yes	☐ No	☐ Not sure
Do you feel your current equipment is comfortable for the animal?	☐ Yes	☐ No	☐ Not sure
In the past year, how many times has your animal been lame?	………… *times*		☐ Not sure
In the past year, has your animal developed harness related wounds?	☐ Yes<br>☐ No<br>☐ Not sure	If "Yes", how often? ☐ Rarely ☐ Occasionally	☐ Often<br>☐ Very often
Do you take any measures to prevent the development of wounds?	☐ Yes<br>☐ No	If "Yes", which measures?<br>………………………………………………	

Comments
