# Supplementary material for: Putting the cart before the horse: mixed-methods participatory investigation of working equid harnessing practices in three selected towns of the Oromia national regional state in Ethiopia
Source: BMC Vet Res. 2024 Mar 22;20:113. doi: 10.1186/s12917-024-03967-3 (PMC10958837; doi:10.1186/s12917-024-03967-3)
Supplement: Supplementary file 2 — Supplementary Material 2 [file 12917_2024_3967_MOESM2_ESM.docx]

**Supplement 2a – Harness components and characteristics**

Supplement 2, Table 1 – Description of the proportion and frequency of harness components and characteristics in a cross-sectional study investigating work-equipment and practices in working equids of three Ethiopian locations.

| **Equipment Components Present**  **Equipment characteristics** | **Frequency** | **Percentage** |
| --- | --- | --- |
| Bit | 366/368 | 99.5% |
| Good condition | 65/366 | 17.8% |
| Correct size/fit | 94/366 | 25.6% |
| Blinkers | 323/368 | 87.8% |
| Dished shape | 102/323 | 31.6% |
| Not touching the eyelashes | 115/323 | 35.6% |
| Reins | 366/368 | 99.5% |
| Freely moving | 364/366 | 99.5% |
| Breast collar | 368/368 | 100% |
| Collar padding | 145/368 | 39.4% |
| Positioned through point of draught | 132/368 | 35.9% |
| Easily adjustable | 10/368 | 2.7% |
| Made from breathable contact materials | 259/368 | 70.4% |
| Neck strap | 211/368 | 57.3% |
| Easily adjustable | 16/211 | 7.6% |
| Made from breathable contact materials | 101/211 | 47.9% |
| Traces | 246/368 | 68.8% |
| Freely moving | 134/246 | 54.5% |
| Swingle tree | 151/368 | 41% |
| Freely moving | 72/151 | 47.7% |
| With traces attaching | 150/151 | 99.3% |
| Functional swingle tree*^1^ | 72/368 | 19.6% |
| Functional traction*^2^ | 17/368 | 4.6% |
| Saddle | 368/368 | 100% |
| Includes a gullet | 203/368 | 55.2% |
| Positioned at base of the withers | 246/368 | 66.8% |
| Wide pressure points | 294/368 | 79.9% |
| Secured tightly in place (girth not loose) | 173/368 | 47% |
| Saddle padding | 368/368 | 100% |
| Padding made from breathable contact materials | 94/368 | 25.5% |
| Functional saddle*^3^ | 33/368 | 9.0% |
| Back band | 86/368 | 23.4% |
| Belly band | 63/368 | 17.1% |
| Girth | 356/368 | 96.7% |
| Easily adjustable | 347/356 | 97.5% |
| Made from breathable contact materials | 248/356 | 69/7% |
| Crupper | 214/368 | 58.2% |
| Made from breathable contact materials | 117/214 | 54.7% |
| Tugs | 361/368 | 98.1% |
| Breeching | 277/368 | 75.3% |
| True breeching | 269/277 | 97.1% |
| False breeching | 8/277 | 2.9% |
| Correct breeching assembly | 60/277 | 21.7% |
| Made from breathable contact materials | 171/277 | 61.7% |
| Functional breeching*^4^ | 60/368 | 16.3% |
| Shafts passing through the centre of gravity | 122/368 | 33.2% |
| Harness is clean | 15/368 | 4.1% |
| Cart | 368/368 | 100% |
| Two-wheeled cart | 368/368 | 100% |
| Pneumatic tyre type | 368/368 | 100% |
| Good tyre inflation | 368/368 | 100% |
| Balanced cart axel | 353/368 | 95.9% |
| **1 Swingle tree is present and moves freely. Traces attach to swingle tree.* | | |
| **2 Adequate position over the point of draught, freely moving traces that attach to a functional swingle tree* | | |
| **3 Adequately positioned, wide pressure points, has gullet, has padding, secured tightly to the animal.* | | |
| **4 Presence of breeching and correctly assembled as either true or false breeching* | | |

Supplement 2, Table 2 - Significant differences between main type of work and the presence and characteristics of the harness components in a cross-sectional study investigating work-equipment and practices in working equids of three Ethiopian locations.

| Harness Characteristics | Effect Direction (n) | Odds Ratio | 95% CI | Wald  p-value | FDR adjusted p-value† |
| --- | --- | --- | --- | --- | --- |
| Presence of breeching |  |  |  | 0.004 | 0.007 |
|  | Goods (93/106) > Taxi (164/232) | 2.97 | 1.56 - 5.66 |  |  |
|  | Goods (93/106) > Water (6/11) | 5.96 | 1.59 - 22.34 |  |  |
| Presence of back-band |  |  |  | <0.001 | <0.001 |
|  | Goods (3/106) < Taxi (77/232) | 0.07 | 0.02 - 0.20 |  |  |
|  | Goods (3/106) < Mixed (6/19) | 0.07 | 0.02 - 0.29 |  |  |
| Presence of girth |  |  |  | 0.013 | 0.017 |
|  | Goods (96/106) < Taxi (230/232) | 0.10 | 0.02 - 0.41 |  |  |
| Presence of belly-band |  |  |  | 0.003 | 0.005 |
|  | Goods (6/106) < Mixed (4/19) | 0.22 | 0.06 - 0.83 |  |  |
|  | Goods (6/106) < Taxi (53/232) | 0.22 | 0.09 - 0.51 |  |  |
| Presence of crupper |  |  |  | <0.001 | <0.001 |
|  | Goods (15/106) < Taxi (186/232) | 0.04 | 0.02 - 0.08 |  |  |
|  | Goods (15/106) < Mixed (10/19) | 0.15 | 0.05 - 0.43 |  |  |
|  | Mixed (10/19) < Taxi (186/232) | 0.28 | 0.11 - 0.70 |  |  |
|  | Taxi (186/232)> Water (3/11) | 9.74 | 2.68 - 35.38 |  |  |
| Presence of blinkers |  |  |  | <0.001 | <0.001 |
|  | Taxi (231/232)> Goods (69/106) | 0.01 | 0.00 - 0.06 |  |  |
|  | Taxi (231/232)> Water (7/11) | 92.60 | 12.63 - 679.07 |  |  |
|  | Taxi (231/232)> Mixed (16/19) | 0.03 | 0.00 - 0.22 |  |  |
| Presence of neck strap |  |  |  | <0.001 | <0.001 |
|  | Taxi (199/232)> Water (0/11) | 136.97 | 7.81 - 2400 |  |  |
|  | Taxi (199/232)> Mixed (7/19) | 0.10 | 0.04 - 0.27 |  |  |
|  | Taxi (199/232)> Goods (5/106) | 0.01 | 0.00 - 0.02 |  |  |
|  | Mixed (7/19)> Goods (5/106) | 0.09 | 0.03 - 0.32 |  |  |
| Presence of traces |  |  |  | <0.001 | <0.001 |
|  | Taxi (231/232)> Water (0/11) | 3549.67 | 135 - 93002 |  |  |
|  | Taxi (231/232)> Mixed (9/19) | 0.01 | 0.00 - 0.04 |  |  |
|  | Taxi (231/232)> Goods (6/106) | 0.00 | 0.00 - 0.00 |  |  |
|  | Mixed (9/19)> Goods (6/106) | 0.07 | 0.02 - 0.23 |  |  |
|  | Mixed (9/19)> Water (0/11) | 20.81 | 1.06 - 407.32 |  |  |
| Appropriate positioning of saddle |  |  |  | <0.001 | <0.001 |
|  | Goods (83/106) > Taxi (135/232) | 2.56 | 1.51 - 4.34 |  |  |
|  | Mixed (18/19)> Taxi (135/232) | 8.88 | 1.64 - 48.08 |  |  |
| Breathability of saddle padding materials |  |  |  | <0.001 | <0.001 |
|  | Taxi (80/232)> Goods (9/106) | 5.42 | 2.63 – 11.14 |  |  |
| Breathability of collar materials |  |  |  | <0.001 | <0.001 |
|  | Taxi (195/232)> Goods (49/106) | 0.17 | 0.10 - 0.28 |  |  |
|  | Taxi (195/232)> Mixed (10/19) | 0.22 | 0.08 - 0.57 |  |  |
|  | Water (5/11)> Taxi (195/232) | 6.16 | 1.79 - 21.21 |  |  |
| Breathability of girth materials |  |  |  | <0.001 | <0.001 |
|  | Taxi (223/230)> Water (2/11) | 89.40 | 19.12 - 417.92 |  |  |
|  | Taxi (223/230)> Mixed (7/19) | 0.03 | 0.01 - 0.08 |  |  |
|  | Taxi (223/230)> Goods (16/96) | 0.01 | 0.00 - 0.02 |  |  |
|  | Mixed (7/19)> Goods (16/96) | 0.30 | 0.11 - 0.87 |  |  |
| Breathability of crupper materials |  |  |  | <0.001 | <0.001 |
|  | Taxi (110/186)> Goods (4/15) | 0.05 | 0.02 - 0.13 |  |  |
|  | Taxi (110/186)> Water (0/3) | 20.75 | 1.20 - 359.59 |  |  |
|  | Taxi (110/186)> Mixed (3/10) | 0.24 | 0.08 - 0.77 |  |  |
|  | Mixed (3/10)> Goods (4/15) | 0.21 | 0.05 - 0.92 |  |  |
| Breathability of breeching materials |  |  |  | <0.001 | <0.001 |
|  | Taxi (137/164)> Goods (27/92) | 0.24 | 0.15 - 0.40 |  |  |
|  | Taxi 137/164)> Water (0/6) | 33.12 | 1.91 - 574.16 |  |  |
| Presence of a functional swingle tree*1 |  |  |  | <0.001 | 0.001 |
|  | Mixed (3/19)> Goods (1/106) | 0.07 | 0.01 - 0.49 |  |  |
|  | Taxi (68/232)> Goods (1/106) | 0.03 | 0.01 - 0.18 |  |  |
| Presence of a functional saddle*2 |  |  |  | 0.007 | 0.010 |
|  | Mixed (5/19)> Goods (1/106) | 0.04 | 0.01 -0.25 |  |  |
|  | Taxi (27/232)> Goods (1/106) | 0.11 | 0.02 - 0.56 |  |  |
| Presence of functional breeching*3 |  |  |  | <0.001 | 0.01 |
|  | Mixed (3/19)> Goods (2/106) | 0.11 | 0.02 - 0.62 |  |  |
|  | Taxi (55/232)> Goods (2/106) | 0.08 | 0.02 - 0.28 |  |  |
| *^1^ *Swingle tree is present and moves freely. Traces attach to swingle tree.*  *^2^ *Adequately positioned, wide pressure points, has gullet, has padding, secured tightly to the animal.*  *^3^ *Presence of breeching and correctly assembled as either true or false breeching.*  *† False discovery rate correction (FDR) using the Benjamini-Hochberg method, adjusting for multiple comparisons.* | | | | |  |

**Supplement 2b – Description of cart-driver questionnaire responses and equipment associations**

Supplement 2, Table 3 - Demographic information of cart-driver questionnaire respondents in a study investigating work-equipment characteristics and practices in working equids of three Ethiopian locations – Bishoftu, Fiche and Shashamene in 2022.

| **Questionnaire Responses** | | **Median** | **Range** |
| --- | --- | --- | --- |
| Driver age (years) | | 28 | 24 - 70 |
| Driver number of dependents | | 3 | 0 - 8 |
| Experience driving equids (years) | | 4 | 1 - 32 |
| Experience with equids (years) | | 4 | 1 - 32 |
| Percentage of income derived from equid (%) | | 100 | 40 - 100 |
|  | |  |  |
| **Questionnaire Responses** | | **Frequency** | **Percentage** |
| Driver gender | |  |  |
|  | *Female* | 4/368 | 1.1% |
|  | *Male* | 364/368 | 98.9% |
| Ownership of equid | |  |  |
|  | *Owner and driver* | 343/368 | 93.2% |
|  | *Driver only* | 25/368 | 6.8% |
| Driver Education | |  |  |
|  | *No formal education* | 28/368 | 7.6% |
|  | *Primary education* | 126/368 | 34.2% |
|  | *Early secondary* | 107/368 | 29.1% |
|  | *Late secondary* | 101/368 | 27.4% |
|  | *Tertiary education* | 5/368 | 1.4% |
|  | *Prefer not to say* | 1/368 | 0.3% |
| Economic comfort level | |  |  |
|  | *Finding it difficult* | 22/368 | 6% |
|  | *Just managing* | 250/368 | 67.9% |
|  | *Comfortable* | 96/368 | 26.1% |
|  | *Very Comfortable* | 0/368 | 0% |
|  | *Prospering* | 0/368 | 0% |
| Level of enjoyment of the profession | |  |  |
|  | *Strongly dislike* | 2/368 | 0.5% |
|  | *Dislike* | 238/368 | 64.7% |
|  | *Neutral* | 126/368 | 34.2% |
|  | *Enjoy* | 2/368 | 0.5% |
|  | *Strongly enjoy* | 0/368 | 0% |

Supplement 2, Table 4 - Significant associations between cart-driver questionnaire responses and characteristics of the harness components in a cross-sectional study investigating work-equipment and practices in working equids of three Ethiopian locations.

| **Questionnaire Variables** | **Equipment Associations** | **Effect Direction (n)** | **Odds Ratio** | **95% CI** | **Wald**  **p-value** | **FDR adjusted p-value†** |
| --- | --- | --- | --- | --- | --- | --- |
| Believing equipment is efficient | Adequacy of saddle position |  |  |  | 0.021 | 0.062 |
|  |  | Believing it is efficient (237/347) > Not believing (9/21) | 2.87 | 1.18 - 7.02 |  |  |
| Income comfort level | Presence of belly band |  |  |  | 0.005 | 0.008 |
|  |  | Higher income comfort (27/96) > lower comfort (34/250) | 2.48 | 1.40 - 4.40 |  |  |
|  | Presence of neck strap |  |  |  | 0.049 | 0.049 |
|  |  | Higher income comfort (145/250) > lower comfort (7/22) | 2.96 | 1.17 - 7.51 |  |  |
| Source of equipment* | Presence of back band | ‘Partly purchased & Homemade' (22/31)> 'Purchased' (59/310) | 10.01 | 4.35 - 25 | <0.001 | <0.001 |
|  | Presence of belly band | ‘Partly purchased & Homemade' (18/31) > 'Purchased' (38/310) | 9.70 | 4.35 - 20 | <0.001 | <0.001 |
|  | Presence of crupper | ‘Partly purchased & Homemade' (28/31)> 'Purchased' (176/310) | 6.20 | 2 - 20 | 0.002 | 0.005 |
|  | Presence of neck strap | ‘Partly purchased & Homemade' (24/31)> 'Purchased' (172/310) | 2.62 | 1.12 - 6.25 | 0.048 | 0.049 |
|  | Presence of traces | ‘Partly purchased & Homemade' (31/31)> 'Purchased' (199/310) | 35.21 | 2.13 - 500 | 0.012 | 0.018 |
|  | Breathability of girth materials | ‘Partly purchased & Homemade' (28/31)> 'Purchased' (203/298) | 4.31 | 1.37 - 14.29 | 0.021 | 0.031 |
|  | Breathability of crupper materials | ‘Partly purchased & Homemade' (20/28)> 'Purchased' (88/176) | 4.48 | 2.08 - 10 | <0.001 | 0.001 |
|  | Breathability of breeching materials | ‘Partly purchased & Homemade' (26/27)> 'Purchased' (131/226) | 6.58 | 2.56 - 20 | 0.001 | 0.004 |
| Driver experience (years) | Traction through the point of draught | Higher experience < traction through the point of draught | coef. = -0.1 | -0.17 to -0.03 | 0.005 | 0.007 |
|  | Shafts passing through the centre of gravity | Higher experience > centre of gravity | coef. = 0.06 | 0.01 to 0.12 | 0.022 | 0.065 |
|  | Equipment having functional traction | Higher experience < functional traction | coef. = -0.29 | -0.6 to -0.06 | 0.034 | 0.137 |
| Source of training about work equipment | Presence of crupper |  |  |  | 0.032 | 0.049 |
|  |  | Learning from 'Harness Maker' (19/24)> 'Other Drivers' (44/89) | 3.89 | 1.33 - 11.32 |  |  |
|  | Presence of neck strap |  |  |  | <0.001 | <0.001 |
|  |  | Learning from 'Intuition & Observation' (167/255) > 'Other Drivers' (32/89) | 3.38 | 2.04 - 5.60 |  |  |
|  | Presence of traces |  |  |  | <0.001 | 0.001 |
|  |  | Learning from 'Intuition & Observation' (185/255)> 'Other Drivers' (43/89) | 2.83 | 1.72 - 4.70 |  |  |
|  |  | Learning from 'Harness Maker' (18/24)> 'Other Drivers' (43/89) | 3.21 | 1.17 - 8.84 |  |  |
|  | Breathability of saddle padding materials |  |  |  | <0.001 | 0.001 |
|  |  | Learning from 'Intuition & Observation' (81/255)> 'Other Drivers' (8/89) | 4.71 | 2.18 - 10.21 |  |  |
|  | Breathability of girth materials |  |  |  | 0.002 | 0.005 |
|  |  | Learning from 'Intuition & Observation' (185/248)> 'Other Drivers' (46/84) | 2.47 | 1.50 - 4.07 |  |  |
|  | Breathability of crupper materials |  |  |  | 0.025 | 0.038 |
|  |  | Learning from 'Harness Maker' (13/19) > 'Intuition and Observation' (72/151) | 3.00 | 1.28 - 7.14 |  |  |
|  | Traction through the point of draught |  |  |  | <0.001 | 0.002 |
|  |  | Learning from 'Intuition & Observation' (106/255)> 'Other Drivers' (16/89) | 3.25 | 1.79 - 5.89 |  |  |
|  |  | Learning from 'Harness Maker' (10/24) > 'Other Drivers' (16/89) | 3.26 | 1.23 - 8.64 |  |  |
|  | Equipment having functional breeching |  |  |  | 0.004 | 0.015 |
|  |  | Learning from 'Other Drivers' (21/89)> 'Intuition & Observation' (31/255) | 2.23 | 1.20 - 4.17 |  |  |
|  |  | Learning from 'Harness Maker' (8/24)> 'Intuition & Observation' (31/255) | 3.61 | 1.43 - 9.14 |  |  |
|  | Equipment having functional girth |  |  |  | 0.016 | 0.065 |
|  |  | Learning from 'Other Drivers' (26/89)> 'Intuition & Observation' (43/255) | 2.04 | 1.16 - 3.57 |  |  |
| **Only associations between ‘Partly purchased & Homemade' and 'Purchased' sources presented*  *† False discovery rate correction (FDR) using the Benjamini-Hochberg method, adjusting for multiple comparisons* | | | | | |  |

**Supplement 2c – Description of focus group participant demographics**

Supplement 2, Table 5 - Demographic information of focus group participants in a study investigating work-equipment characteristics and practices in working equids of three Ethiopian locations – Bishoftu, Fiche and Shashamene in 2022.

| Demographic Variable | Frequency of Participants | Percentage of Participants |  |
| --- | --- | --- | --- |
| Age group |  |  | |
| *18-20* | 2/87 | 2.3% | |
| *21-30* | 6/87 | 6.9% | |
| *31-40* | 56/87 | 64.4% | |
| *41-50* | 17/87 | 19.5% | |
| *51-60* | 3/87 | 3.5% | |
| *61-70* | 2/87 | 2.3% | |
| *>70* | 1/87 | 1.2% | |
| Gender |  |  | |
| *Male* | 87/87 | 100% | |
| Level of education |  |  | |
| *No formal education* | 9/84 | 10.7% | |
| *Primary education* | 19/84 | 22.6% | |
| *Early secondary* | 39/84 | 46.4% | |
| *Late secondary* | 15/84 | 17.9% | |
| *Tertiary education* | 2/84 | 2.4% | |
| Occupation relating to working equid |  |  | |
| *Owner and driver* | 77/87 | 88.5% | |
| *Veterinary professional* | 1/87 | 1.2% | |
| *Harness maker* | 9/87 | 10.3 | |
| Occupation relating to working equid is the primary occupation | 87/87 | 100% | |
| Source of training (owners and drivers) |  |  | |
| *No formal training* | 62/77 | 80.6% | |
| *Other drivers* | 4/77 | 5.2% | |
| *Local NGO* | 11/77 | 14.3% | |
| Source of training (harness makers) |  |  | |
| *No formal training* | 8/9 | 88.9% | |
| *Local NGO* | 1/9 | 11.1% | |
| Economic comfort level |  |  | |
| *Finding it difficult* | 5/86 | 5.8% | |
| *Just managing* | 49/86 | 57% | |
| *Comfortable* | 32/86 | 37.2% | |
| *Very comfortable* | 0/86 | 0% | |
| *Prospering* | 0/86 | 0% | |
